# Construction of prognosis model of pyroptosis gene and characterization of tumor microenvironment infiltration in cervical cancer

**DOI:** 10.1097/MD.0000000000031599

**Published:** 2022-11-18

**Authors:** Min Zhang, Peng Wu

**Affiliations:** a Department of Gynecology, Zhejiang Xiaoshan Hospital, Hangzhou, China.

**Keywords:** cervical cancer, overall survival, prognosis, pyroptosis, tumor microenvironment

## Abstract

Pyroptosis has been demonstrated in recent years to be an inflammatory form of programmed cell death. However, the prognostic evaluation of cervical cancer (CESC) to pyroptosis is insufficient and their correlations with prognosis remain unclear. In this study, we identified 15 differentially expressed pyroptosis-related genes between tumor samples and normal samples. By using Cox regression analysis, a 3-gene risk signature was built and classified all CESC patients in the cancer genome atlas cohort into a low-risk or high-risk group. CESC patients in the low-risk group showed significantly higher survival probabilities than those in the high-risk group (*P* < .05). And the risk score was found to be an independent factor for predicting the overall survival of CESC patients. Besides, based on 33 pyroptosis-related genes, all CESC cases could be divided into 3 clusters with consensus clustering analysis. We characterized and analyzed the characteristics of tumor microenvironment infiltration in different clusters. Our findings provide a foundation for future research targeting pyroptosis and its immune microenvironment to improve prognosis in CESC patients.

## 1. Introduction

Cervical cancer (CESC) is the fourth most common cancer worldwide and also the main cause of cancer-related deaths in women.^[1]^ CESC is also a highly malignant gynecological tumor that has a poor prognosis. Traditional interventions have failed to improve the overall survival period of patients due to high tumor recurrence after treatment or late diagnosis.^[2]^ The development of a specific prognostic model is important if we need to improve treatment strategies. Cervical cancer is the 4^th^ most prevalent cancer among women. In developing countries, it is the leading cause of cancer-associated mortalities.^[3]^ The median age for patients diagnosed with cervical cancer is 49 years. In general, cervical cancer lowers the quality of life of the affected persons. It has been established that prolonged infection with human papillomavirus (HPV) types 16 and 18 is a risk factor for cervical cancer.^[4]^ Prophylactic vaccines against high-risk HPV types minimize the risk of developing cervical cancer. However, due to the limitations associated with HPV vaccines, reliable therapeutic options for cervical cancer, particularly recurrent or advanced tumors are required.^[5]^ Current therapeutic options include surgical removal of the tumors based on the FIGO staging system, incorporated with chemo or radiotherapies.^[6,7]^ As for recurrent cervical cancer, bevacizumab in combination with other therapies can significantly prolong patients’ survival time.^[8]^ In addition, immunotherapy is a viable option for cervical cancer treatment.

Pyroptosis referred to as cellular inflammatory necrosis, is considered to be gasdermin-mediated programmed necrotic cell death.^[9]^ Triggered by certain inflammasomes, pyroptosis relies on the cleavage of gasdermin D (GSDMD) and activation of inactive cytokines.^[10]^ The correlation between pyroptosis and cancer is extremely complicated. Although pyroptosis can inhibit the oncogenesis and progression of tumors, it also develops a microenvironment delivering nutrients for cancer and accelerating cancer growth.^[11]^ Increasing studies have demonstrated the effect of pyroptosis on tumor cell proliferation, invasion, and metastasis, thus affecting the prognosis of cancer.^[12,13]^

In previous research, Shi et al^[14]^ found that Caspase 1 (CASP1) and caspase 4/5 could specifically cleave GSDMD and that the cleaved form of GSDMD is necessary for pyroptosis; these findings were subsequently confirmed by He et al.^[15]^ Actually, in cervical cancer, breast cancer and gastric cancer, pyroptosis plays an important role.^[16]^ Studies have shown that pyroptosis can have the potential to become a diagnostic marker of cancer and contribute to the prevention and treatment of cancer. In some cases, pyroptosis is undoubtedly beneficial to human health, especially for cancer treatment. As a kind of cell death, pyroptosis can inhibit the occurrence and development of cancer. Some studies also have shown that chemotherapeutic drugs, reagents, natural products and targeted therapy drugs can cause pyroptosis and death of many cancers. Therefore, pyroptosis is expected to become a new target for cancer treatment.

In the present study, we aimed to build a scoring model (that produced the risk score) by classifying CESC patients based on pyroptosis-related regulators to predict prognosis and guide clinical treatment. The risk score can be determined by constructing a pyroptosis-related model using the Cox regression analysis method. This model can predict prognosis. On this basis, we clustered 296 CESC patients according to pyroptosis-related genes and identified 3 pyroptosis-related clusters that were related to prognosis and immune infiltration. Our findings indicate the potential connection between pyroptosis, prognosis, the immune microenvironment of CESC patients.

## 2. Materials and methods

### 2.1. Sources of cervical cancer datasets and preprocessing

The expression data and clinical follow-up data of cervical cancer (CESC) counts were obtained from UCSC Xena. As to datasets in the cancer genome atlas (TCGA), RNA sequencing data (counts value) of gene expression were downloaded from UCSC. Patients without survival information were removed from further analysis. A total of 299 samples were downloaded, 296 primary cancer samples and 3 normal samples were extracted for follow-up analysis. All of the clinical information used in this study are publicly available in the GEO database. Download address: https://xenabrowser.net/datapages/.

First, convert the Ensembl ID in the expression profile into gene symbol, then extract the coding gene expression profile, average the expression value of the same gene, and rank the cancer samples with the normal samples to obtain the coding gene expression profile. The expression data of 33 pyroptosis genes were extracted.

### 2.2. Identify pyroptosis-related genes

A total of 33 pyroptosis-related genes were obtained from prior reviews,^[17–20]^ we obtained 33 genes (*ELANE, GSDMC, NLRC4, CASP5, NLRP3, IL6, AIM2, CASP1, CASP3, CASP4, CASP6, CASP8, CASP9, GPX4, GSDMA, GSDMB, GSDMD, GZMA, GZMB, IL18, IL1B, NLRP1, NLRP2, NLRP6, NLRP7, NOD1, NOD2, PLCG1, PRKACA, PYCARD, SCAF11, TIRAP, TNF*) that related closely to CESC as pyroptosis-related genes.

### 2.3. Differentially expressed pyroptosis-related genes

We calculated the difference in gene expression between normal samples and cancer samples and the difference in the expression of 33 pyroptosis genes by *t*-test, with a threshold of *P* < .05.

### 2.4. Univariate and multivariate cox survival analyses

We used univariate Cox regression and multivariate Cox regression analyses for overall survival in datasets described earlier. *P*-value < 0.05 and 0.1 were used as the statistical boundary for univariate and multivariate separately. The results of multivariate prognostic analysis for pyroptosis-related subgroups were acquired by application of the “survival” package.

### 2.5. The establishment of a scoring model and prognostic analysis

We established an efficient prediction model using Cox analysis. Overall survival was then used to derive the most useful predictive features from the datasets.


$$\begin{array}{l} Risk\;score=\sum_{i=1}^{k}\beta_{i}P_{i}, \end{array}$$

where *k*, *β*_i_, *P*_*i*_ represented the number of signature genes, the coefficient index, and the gene expression level, respectively. We used Kaplan–Meier survival curves to identify the ability of the model to distinguish different subtypes of patients and time-dependent receiver operating characteristic curves (ROC curves) to determine the efficiency of the model.

### 2.6. Consensus clustering

Consensus clustering was applied to identify distinct pyroptosis-related patterns relating to the expression of pyroptosis regulators by the k-means method. The number of clusters, and their stability, were determined by the consensus clustering algorithm using the “ConsensuClusterPlus” package.^[21]^ We performed 1000 times repetitions, the proportion of samples extracted in each round of iteration is 80%, and the optimal number of clusters is determined by cumulative distribution function to guarantee the stability of our classification.^[22]^

### 2.7. Tumor microenvironment (TME) cell infiltration

We applied the immunecell immunity identifier (ImmuCellAI) method (http://bioinfo.life.hust.edu.cn/web/ImmuCellAI/), to quantify the relative abundance of each cell infiltration in the tumor microenvironment. ImmuCellAI is the first tool focusing on the prediction of T cell subtype abundance. Based on the enrichment score of characteristic gene set rather than the traditional deconvolution method, single sample gene set enrichment analysis is used to calculate the score of the ratio of various immune cell specific expression gene sets in the sample and immune cell reference data set, to obtain the abundance of 24 immune cells in the sample. The results showed that ImmuCellAI is not only much more than the existing tools in the number of predictable T cell subtypes, but also significantly better than the existing tools in the accuracy of prediction results.^[23]^

### 2.8. Gene set variation analysis (GSVA)

We performed gene set variation analysis (GSVA) enrichment analysis by the“GSVA” R packages.^[24]^ and then downloaded “c2.cp.kegg.v7.4.symbols” from the MSigDB database to carry out GSVA analysis. An adjusted *P* < .05 was considered to indicate statistical significance between different subgroups.

### 2.9. Statistical analysis and cut-off value

Correlation coefficients were computed by Spearman’s and distance correlation analyses. Log-rank tests were utilized to identify the significance of differences in survival curves. The cutoff value of high-and low-risk scores was median from the “survival” package. ROC curves were derived using the “timeROC” packages. Gene expression data and all statistical analyses were carried out in R 3.6.0.

## 3. Results

### 3.1. Identification of differentially expressed pyroptosis-related genes between normal and tumor tissues

**Figure 1.**
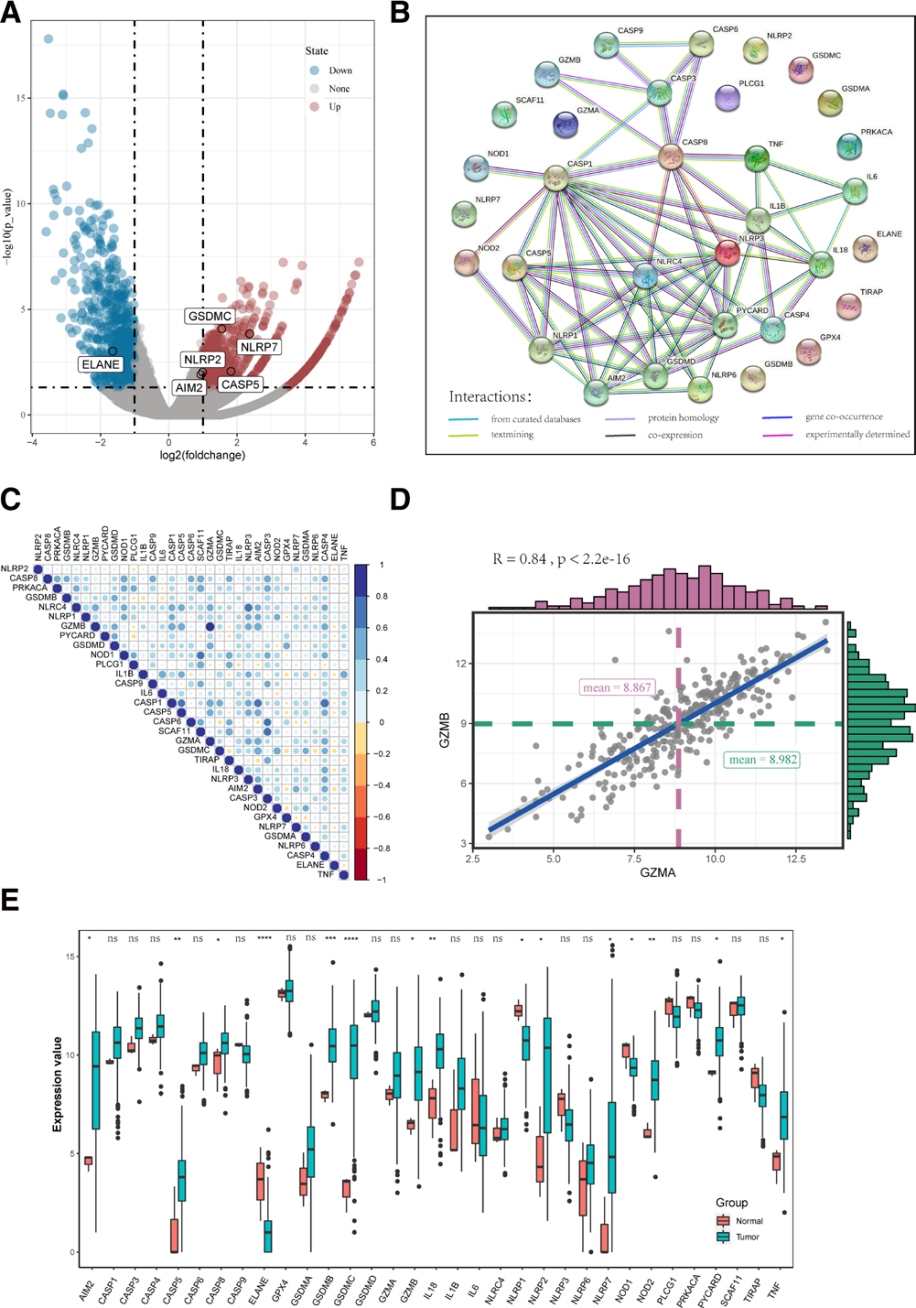
Expression and interaction of 33 pyroptosis genes in cervical cancer A. Volcano map of gene differential expression between cancer samples and normal samples in cervical cancer. B. Protein interaction network of 33 pyroptosis genes. C. Correlation between the expression of 33 pyroptosis genes in cervical cancer samples. D. Scatter plot of correlation trend between GZMA and GZMB. E. The box diagram shows the transcriptome expression status of 33 pyroptosis genes between normal samples and cancer samples.

The 33 pyroptosis-related genes expression levels were compared and TCGA data from 3 normal and 296 tumor tissues, and we identified 15 differentially expressed pyroptosis-related genes (*P* < .05). The difference of gene expression between normal samples and cancer samples was calculated by *t*-test (Fig. 1A) (red: up-regulated; blue: down-regulated). To further explore the interactions of these pyroptosis-related genes, we conducted a protein-protein interaction (PPI) analysis, and the results are shown in Figure 1B. The minimum required interaction score for the PPI analysis was set at 0.9 (the highest confidence), and we determined that, CASP1 was hub genes. Among them, 15 differentially expressed pyroptosis-related genes between normal and tumor tissues. The correlation network containing all pyroptosis-related genes is presented in Figure 1C. The scatter plot (Fig. 1D.) shows the trend of correlation between GZMA and GZMB. Finally, the boxplot of Figure 1E. shows the transcriptome expression status of 33 pyroptosis-related genes between normal samples and cancer samples.

### 3.2. Development of a prognostic risk model in the TCGA cohort based on pyroptosis-related genes

**Figure 2.**
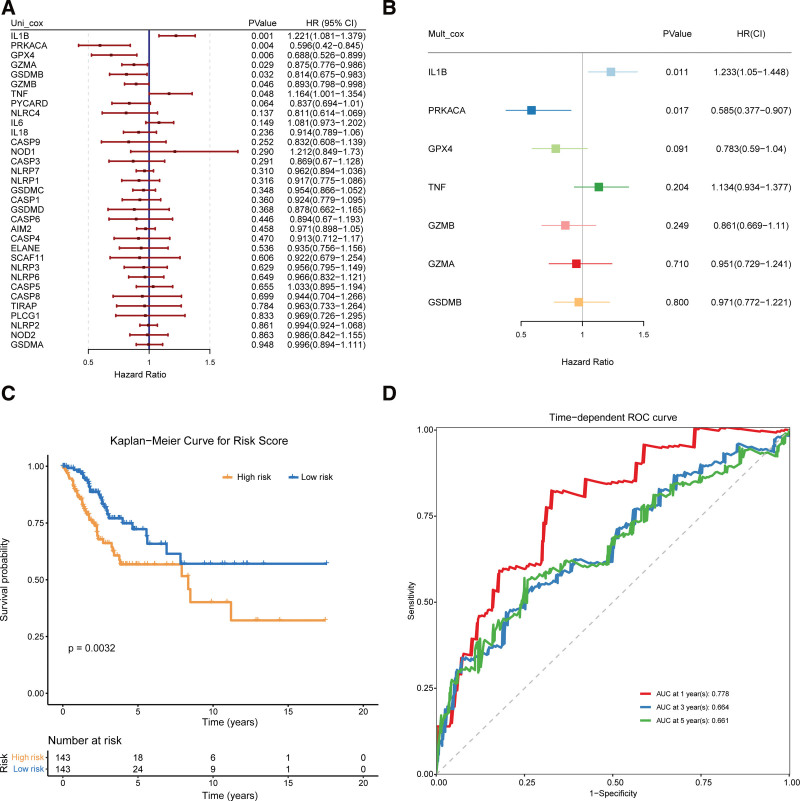
Construction of prognosis model by pyroptosis gene. A. Univariate Cox regression analysis was used to study the relationship between pyroptosis and cervical cancer, and 7 pyroptosis genes related to cervical cancer were revealed (*P* < .05). B. The risk coefficient was obtained by multivariate Cox regression analysis, and the risk score of cervical cancer samples was calculated. C. The survival curves of high-risk and low-risk groups were compared by log rank test. D. ROC curve was drawn for the prognosis model to evaluate the prediction effect. ROC curve = receiver operating characteristic curve.

A total of 286 CESC samples were matched with the corresponding patients who had complete survival information. Univariate Cox regression analysis was used for primary screening of the survival-related genes. In univariate Cox regression analysis, from the expression and survival data of 33 pyroptosis-related genes, it was found that 7 differentially expressed pyroptosis-related genes(*IL1B, PRKACA, GPX4, GZMA, GSDMB, GZMB*, and *TNF*) had significant prognosis that met the criteria of *P* < .05 (Fig. 2A). After further multivariate regression, cox regression analysis, 3 pyroptosis-related genes (*IL1B, PRKACA, and GPX4*) met the criteria of *P* < .1, among them, IL1B was associated with increased risk with HRs > 1, while the other 2 genes (*PRKACA and GPX4*) were protective genes with HRs < 1(Fig. 2B), and the corresponding risk coefficients were obtained.

A risk scoring model is established based on the 3 risk factors. *β* Coefficient from Cox analysis as weight. The risk score was calculated as follows: Risk score = 0.21*IL1B + (-0.54)* PRKACA + (-0.24)*GPX4. Then we establish a risk scoring model, calculate the risk scores of all samples according to the model. Based on the median score calculated by the risk score formula, 286 patients were divided into the low-risk and the high-risk subgroups. We used Kaplan to Meier survival curves to identify the ability of the model to distinguish different subtypes of patients. The prognosis of low-risk group was significantly better than that of high-risk group. A notable difference in survival probability was detected between the low-risk and high-risk groups (*P* < .05, Fig. 2C). Time dependent receiver operating characteristic (ROC) analysis was applied to evaluate the sensitivity and specificity of the prognostic model. ROC curve analysis showed that our model had good predictive efficacy (AUC = 0.778 for 1-year, 0.664 for 3-year, and 0.661 for 5-year survival) (Fig. 2D). At the expression level, these 3 genes were able to help us distinguish low risk samples from high risk samples in patients with cervical cancer. Our analysis showed that the expression levels of pyroptosis-related genes were related to CESC, thus suggesting they may reflect different traits in patients.

### 3.3. CESC classification pattern based on the pyroptosis-related genes

Based on the expression levels of 33 pyroptosis-related genes, we identified 3 different regulation patterns by using the unsupervised clustering method, including 114 cases in pyroptosis-related cluster 1, 52 cases in cluster 2 and 120 cases in cluster3.

**Figure 3.**
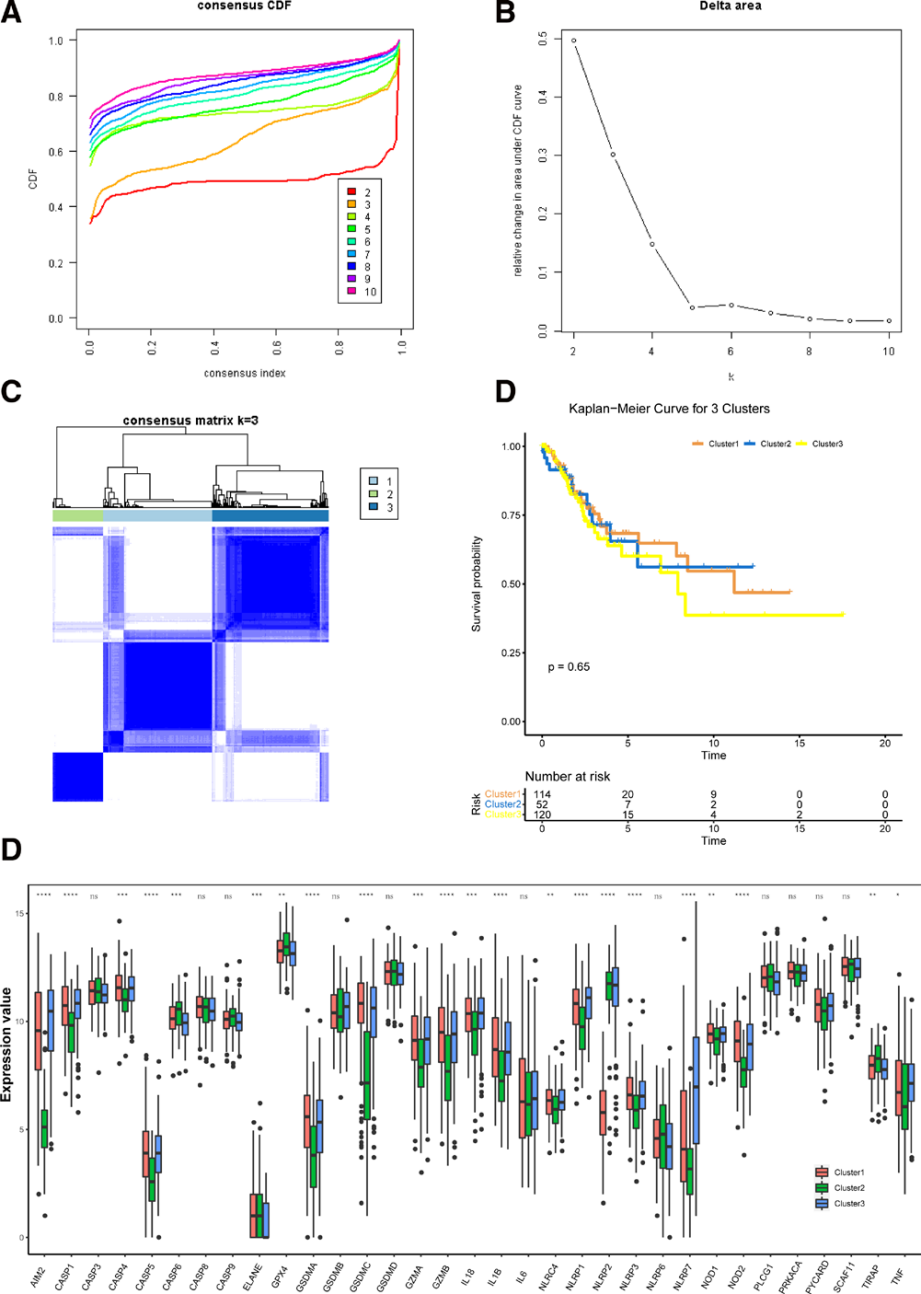
Unsupervised clustering of 33 pyroptosis genes. To identify 3 different pyroptosis pattern subtypes in cervical cancer. A. K = consistency clustering cumulative distribution function (CDF) of 2-10. B. Relative change of area under CDF curve at k = 2-10. C. When k = 3, the clustering heat map of samples. D. Survival analysis of 3 pyroptosis subtypes. E. Differential expression of 33 cell death genes in 3 subtypes. CDF = cumulative distribution function.

To explore the connections between the expression of the 33 pyroptosis-related genes and CESC subtypes, we performed a consensus clustering analysis with all CESC patients in the TCGA cohort. Euclidean distance is used to calculate the similarity distance between samples, and K-means is used for clustering. The number of repeated sampling is 1000, and the proportion of samples extracted in each round of iteration is 80%. The optimal number of clusters is determined by cumulative distribution function cumulative distribution function (Fig. 3A). It can be seen from the figure that increasing the clustering variable (k) from 2 to 10 (Fig. 3B), we found that when k = 3, the intragroup correlations were the highest and the intergroup correlations were low, indicating that the CESC patients could be well divided into 3 clusters based on the 33 pyroptosis-related (Fig. 3C). It can be seen from the figure that 286 tumor samples are assigned to 3 categories. The overall survival probability was also compared between the 3 clusters, no obvious differences were found (*P* = .65, Fig. 3D), but there was a significant difference in the transcriptional profiles of 33 pyroptosis genes (Fig. 3E).

### 3.4. Differences in TME infiltration and clinical characteristics between three pyroptosis-related subtypes

**Figure 4.**
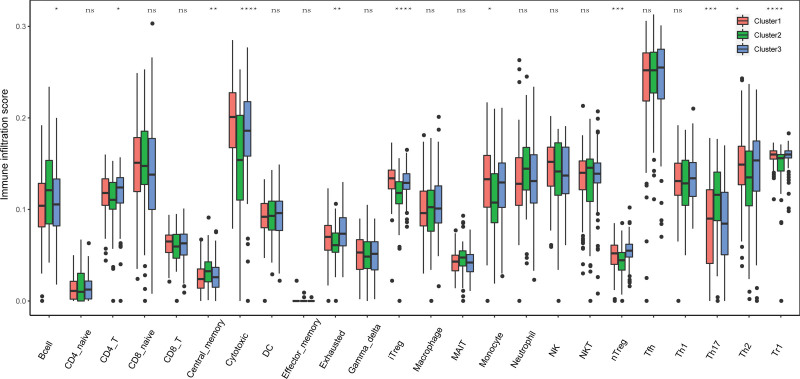
Diversity of immune microenvironment characteristics in different Pyroptosis-Related Genes modes. The abundance of infiltrating immune cells in each immune microenvironment in the 3 pyroptosis-related genes patterns was different.

Based on the functional analyses, we further compared the enrichment scores of different types of immune cells between the 3 clusters by employing the single-sample gene set enrichment analysis (ssGSEA). The samples of cluster1 in the TCGA cohort (Fig. 4) exhibited higher levels of immune cell infiltration, particularly cytotoxic, iTreg, nTreg than the other 2 clusters Figure 4 shows the difference of immune infiltration scores among the 3 clusters. We found that these 3 regulatory patterns had different TME cell infiltration characteristics.

### 3.5. Identification of a CESC classification pattern mediated by 33 pyroptosis-related genes

**Figure 5.**
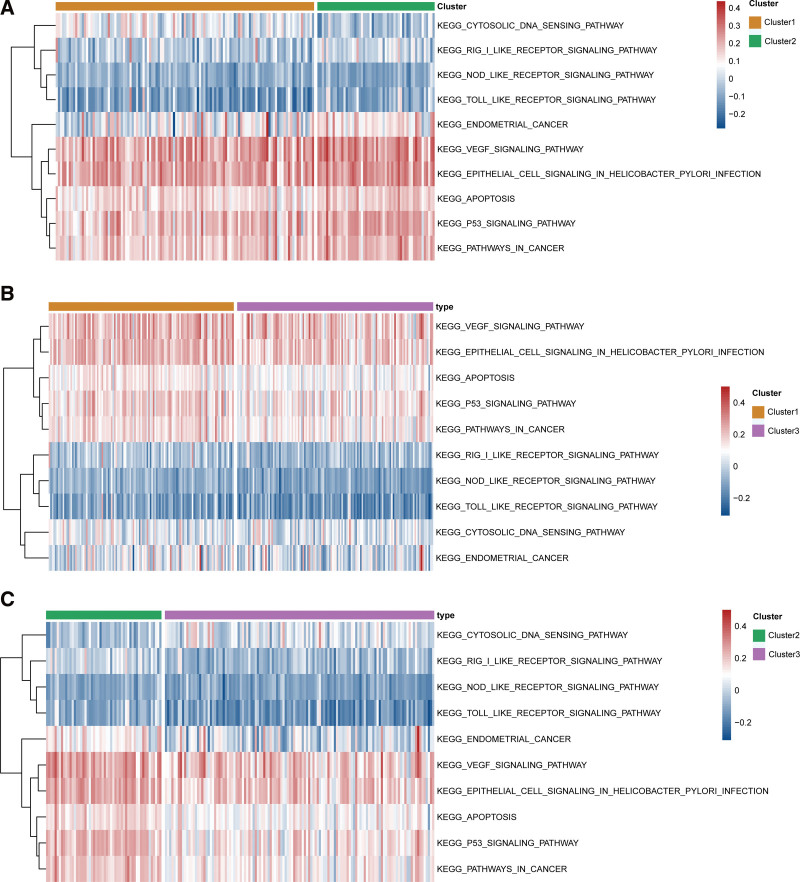
Diversity of potential biological functional characteristics among 3 pyroptosis-related genes modes. A. The difference of KEGG pathway enrichment score between mode 1 and mode 2. B. The difference of KEGG pathway enrichment score between mode 1 and mode 3. C. The difference of KEGG pathway enrichment score between mode 2 and mode 3.

To explore the differences in biological behavior between these 3 patterns, we performed GSVA enrichment analysis using the “GSVA” package (Fig. 5). In nonparametric and unsupervised methods, GSVA is usually used to estimate the changes of pathways and biological processes in the samples of expression data sets. Download the “C2. Cp.kegg. V7.4. Symbols” gene set from msigdb database for gsva analysis.*P*-value < 0.05 was considered statistically significant.

## 4. Discussion

The microenvironment composition of cervical cancer will affect the progress of cervical cancer, but the potential mechanism is still unclear. In the previous study of different solid tumors, the molecular characteristics of these tumors were described to be related to different clinical prognosis, which is helpful to improve clinical efficacy and whole course medical management, through individualized treatment drugs and measures. Pyroptosis is a new type of programmed cell death,^[18]^ which has been found to play a dual role in the development and treatment mechanism of tumors in recent years. On the 1 hand, normal cells are stimulated by a large number of inflammatory factors released by scorching, leading to their transformation into tumor cells.^[25]^ On the other hand, promoting tumor cell pyroptosis may be a new therapeutic target. In CESC patients, it is not clear how the pyroptosis death related genes interact and whether they are related to the survival time and prognosis of patients.

In this study, we analyzed the expression and survival data of 33 pyroptosis-related genes, and found that 7 differentially expressed pyroptosis-related genes had significant prognosis. According to the expression and survival data of 7 pyroptosis-related genes with significant prognosis, a multivariate COX proportional risk regression model was used to obtain 3 pyroptosis-related genes risk factors. The risk scoring model is established based on 3 risk factors. By calculating the risk score of the sample, it was divided into 2 groups, the high risk group and low risk group. It was found that the prognosis effect of the low risk group was significantly higher than that of the high risk group. By drawing ROC curve, it is further proved that risk score is a good indicator of 1-year survival rate, 3-year survival rate and 5-year survival rate of cervical cancer patients. The immune cell infiltration and activated pathways in the different clusters were compared, and we found that these 3 regulatory patterns had different TME cell infiltration characteristics.

Our study was aimed to classify patients with CESC into subtypes, identify differentially expressed pyroptosis-related genes and build a prognostic model, and link pyroptosis with patient prognosis. There is also a need for excavating or building more cervical cancer immunotherapy data. Moreover, our model should be validated further by performing both in vitro and in vivo experiments to better evaluate the relationship between the risk score and pyroptosis of cells after infection. This poses challenges for our further research, and is also the direction that we need to continue in-depth research.

## Author contributions

**Data curation:** Min Zhang.

**Formal analysis:** Min Zhang, Peng Wu.

**Investigation:** Min Zhang.

**Resources:** Min Zhang.

**Methodology:** Peng Wu.

**Supervision:** Peng Wu.

**Writing – original draft:** Min Zhang.

**Writing - review & editing:** Peng Wu.
